# Efficacy of propofol-supplemented cardioplegia on biomarkers of organ injury in patients having cardiac surgery using cardiopulmonary bypass: A protocol for a randomised controlled study (ProMPT2)

**DOI:** 10.1177/02676591231157269

**Published:** 2023-02-16

**Authors:** Rachael Heys, Gianni D Angelini, Katherine Joyce, Helena Smartt, Lucy Culliford, Rachel Maishman, Samantha E de Jesus, Costanza Emanueli, M-Saadeh Suleiman, Prakash Punjabi, Chris A Rogers, Ben Gibbison

**Affiliations:** 1 Bristol Trials Centre, Bristol Medical School, University of Bristol, Bristol, UK; 2 Bristol Heart Institute, University of Bristol, Bristol, UK; 3 National Heart and Lung Institute, Hammersmith Hospital, London, UK; 4 Department of Anaesthesia, University Hospitals Bristol and Weston NHS Foundation Trust, Bristol, UK

**Keywords:** cardiac surgery, cardiopulmonary bypass, cardioplegia, ischemia, reperfusion, propofol, randomised controlled trial

## Abstract

**Introduction:**

Cardiac surgery with cardiopulmonary bypass and cardioplegic arrest is known to be responsible for ischaemia and reperfusion organ injury. In a previous study, ProMPT, in patients undergoing coronary artery bypass or aortic valve surgery we demonstrated improved cardiac protection when supplementing the cardioplegia solution with propofol (6 mcg/ml). The aim of the ProMPT2 study is to determine whether higher levels of propofol added to the cardioplegia could result in increased cardiac protection.

**Methods and Analysis:**

The ProMPT2 study is a multi-centre, parallel, three-group, randomised controlled trial in adults undergoing non-emergency isolated coronary artery bypass graft surgery with cardiopulmonary bypass. A total of 240 patients will be randomised in a 1:1:1 ratio to receive either cardioplegia supplementation with high dose of propofol (12 mcg/ml), low dose of propofol (6 mcg/ml) or placebo (saline). The primary outcome is myocardial injury, assessed by serial measurements of myocardial troponin T up to 48 hours after surgery. Secondary outcomes include biomarkers of renal function (creatinine) and metabolism (lactate).

**Ethics and Dissemination:**

The trial received research ethics approval from South Central – Berkshire B Research Ethics Committee and Medicines and Healthcare products Regulatory Agency in September 2018. Any findings will be shared though peer-reviewed publications and presented at international and national meetings. Participants will be informed of results through patient organisations and newsletters.

**Trial Registration:**

ISRCTN15255199. Registered in March 2019.

## Introduction

More than 32,000 cardiac surgery operations are carried out each year in the UK. To allow surgeons to operate on a still heart in a bloodless field; a cross-clamp is applied to the aorta and the heart is isolated from the rest of the body using a cardiopulmonary bypass (CPB) circuit. Cardioplegia solution is infused directly into the coronary arteries to render the heart flaccid and motionless. However, aortic cross-clamping can cause global ischemia of the myocardium, making it susceptible to reperfusion injury (RPI) when the heart is restarted and blood supply returns.^1,2^ Upon reperfusion, the renewed availability of oxygen leads to a surge in the formation of mitochondrial reactive oxygen species (ROS) and calcium ion loading, both of which cause cardiac muscle cell death.^3,4^ Poor myocardial protection during heart surgery can result in loss of myocardium and subsequent heart failure. Whilst current cardioplegic techniques are effective, improved protection is required in some situations.^1,3,5^

Propofol is widely used during cardiac surgery, mostly as an anaesthetic.^6^ In addition to its anaesthetic effect, propofol has also been shown to be cardioprotective during surgery, both in animal models^7–9^ and humans.^10^ The animal studies included a clinically relevant pig model^9^ which used a blood concentration of propofol of around 3.7 mcg/ml (20 μM). Leading on from this, the PROMPT trial investigated the supplementation of cardioplegia solution with propofol in patients undergoing coronary artery bypass graft (CABG) or aortic valve replacement (AVR) surgery.^10,11^ The addition of propofol was found to be cardioprotective, with an average of 15% lower cardiac troponin T (cTnT) release.^10^ The effect was greater in the CABG patients than in those undergoing AVR. However, the PROMPT trial used a relatively low dose of propofol (6 mcg/ml) to minimise the risk to participants. Having observed no harms attributable to propofol supplemented cardioplegia, the next step is to explore the efficacy of a higher dose of propofol. The placebo that was used in PROMPT (Intralipid, Fresenius Kabi, Uppsala. Sweden) is also potentially cardioprotective.^12–14^ Therefore, in this study saline will be used instead as a placebo.

## Methods and analysis

### Aims and objectives

The ProMPT2 study aims to compare the effect of two different concentrations of propofol in the cardioplegia solution versus placebo (saline) in adults undergoing isolated CABG on: biomarkers of cardiac injury and metabolism; frequency of serious adverse events; and Quality of Life (QoL) in follow up. The study also aims to investigate the effects of the different concentrations of propofol on oxidative stress.

### Study design

The ProMPT2 study is a multi-centre, parallel, three-group, randomised controlled trial (RCT) with blinding, comparing the efficacy and safety of different concentrations of propofol in the cardioplegia solution versus placebo.

The ProMPT2 study is designed in two phases; Phase one will set-up and recruit in two or more NHS surgical centres (University Hospitals Bristol and Weston NHS Foundation Trust and Imperial College Healthcare NHS Trust) with integrated monitoring and feedback to maximise recruitment over a 12 month period; and Phase two will continue recruitment using the optimum methods of recruitment established in Phase one, opening additional centres if required. Progression from Phase one to Phase two will be contingent on meeting the following criteria;

(a) monitoring of early outcome data by the Data Monitoring and Safety Committee (DMSC) suggested that the interventions are safe, and
(b) sufficient participants are recruited to meet the target sample size, specifically that a minimum of 72 participants are recruited within 12 months or the study team are able to provide a plan to make up a recruitment shortfall that satisfies the funder.

### Study population and eligibility criteria

Patients will be eligible for the study if all of the following apply;

(1) Aged ≥18 years;
(2) Having elective or urgent isolated CABG with CPB;
(3) Able to give informed consent;
(4) Women only: negative pregnancy test, or be surgically sterile or post-menopausal for >12 months.

Patients will be ineligible for the study if any of the following apply;

(1) Have had previous cardiac surgery;
(2) Require a planned concomitant procedure;
(3) Require an emergency or salvage operation;
(4) Have long-term steroid therapy (taking tablets on a daily basis for at least 1 month prior to surgery);
(5) Have pre-operative estimated glomerular filtration rate ≤30 mls/min/1.73 m^2^;
(6) Have current congestive heart failure;
(7) Have a left ventricular (LV) ejection fraction <30% (i.e. poor LV function);
(8) Are allergic to peanuts, eggs, egg products, soybeans or soy products;
(9) Are already participating in another interventional clinical study;
(10) Are a prisoner;
(11) Are taking immuno-suppressants (e.g. methotrexate or azathioprine);
(12) Are known to have a cardiac troponin T cTnT level >500 ng/L (or cTnI level >600 ng/L) in the last 4 days (prior to eligibility check);
(13) Women only: breast feeding.

### Patient approach and consent

Potential participants will be identified from clinic and operating theatre waiting lists and Participating Identification Centres (PICs). All potential participants will be sent or given an invitation letter and Patient Information Leaflet (PIL). A member of the local research team will discuss the study with potential participants, answer any questions and obtain informed consent. Potential participants can give written consent in person at their hospital visit or by video or telephone call. Participants who consent via video call or telephone may be guided through the process of completing the consent form by the local research team who will countersign on receipt.

**Figure 1:**
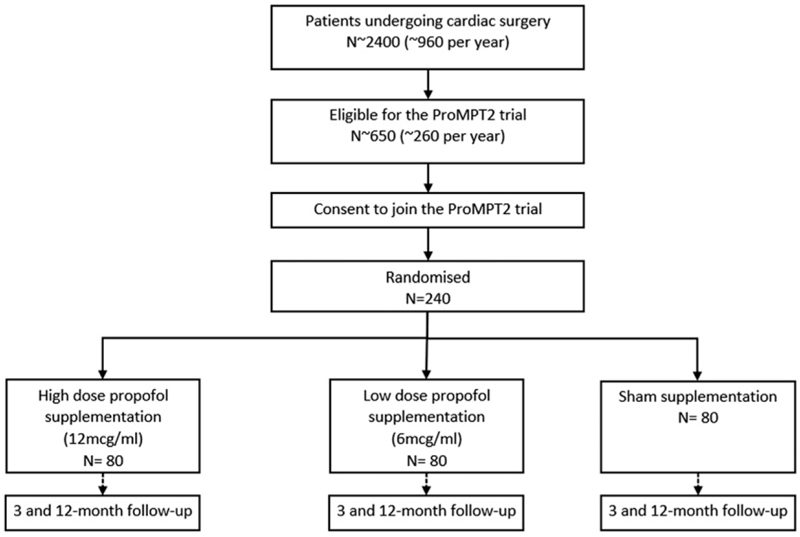
Study schema.

Figure 1 shows the expected pathway through the trial for the participants.

### Study interventions

Eligible patients who give informed consent, will be randomly allocated in a 1:1:1 ratio to receive one of the following:

#### Placebo

Blood cardioplegia with placebo supplementation (normal saline 0.9% weight/volume sodium chloride, NaCl)

#### Propofol low dose

Blood cardioplegia with low dose (6mcg/ml; 33.7 μM) propofol supplementation

#### Propofol high dose

Blood cardioplegia with high dose (12mcg/ml; 67.3 μM) propofol supplementation

We will use Lipuro^®^ 1% propofol emulsion for injection or infusion, B. Braun as it can be administered with saline rather than Intralipid^®^ (which may be cardioprotective) or glucose solution. The pharmacokinetics of propofol given in cardioplegia is included in supplementary material. Study medication (propofol/saline) will be labelled and supplied by the local hospital pharmacy department in accordance with Good Clinical Practice (GCP) and stored in a restricted access area at room temperature, below 25°C. Propofol 1% will be diluted 1 in 5 with 0.9% saline (as recommended by the manufacturers) to achieve a working solution of 2000 mcg/ml.

#### Cardioplegia delivery

**Figure 2:**
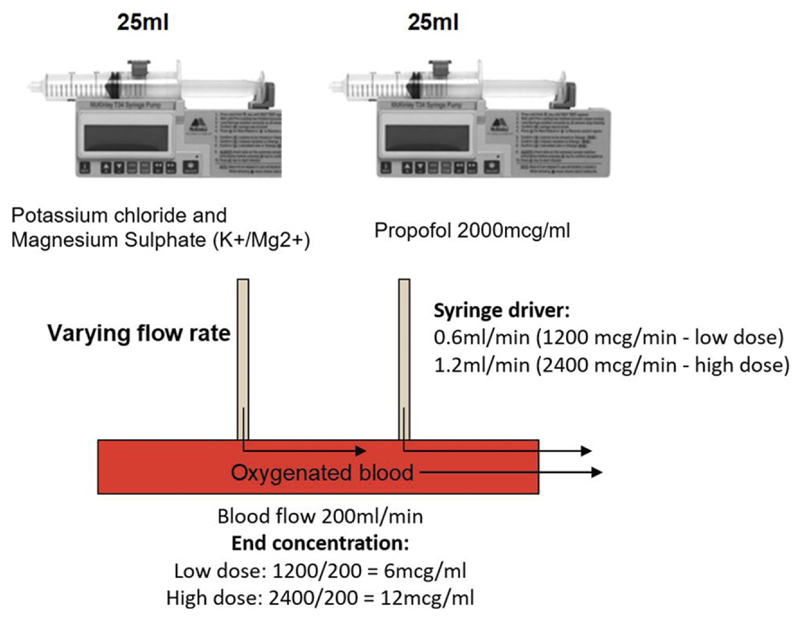
Propofol supplementation of cardioplegia. A roller pump will draw oxygenated blood. Both the cardioplegia solution and intervention will be added downstream of the blood oxygenator. Cardioplegia solution will be delivered into the blood stream via a 60 mL syringe driver (left). An additional syringe driver (right) will be used to deliver the intervention (propofol or saline) into the blood stream.

Warm blood cardioplegia with intermittent antegrade delivery will be used for all groups, as described by Calafiore et al.^15^ and as used in the PROMPT trial.^10,11^ Delivery will be according to the preference of the operating surgeon. The standard cardioplegia composition is described in supplementary material. The study intervention (diluted propofol solution or saline) will be added to the cardioplegia by attaching an additional syringe pump downstream of the blood oxygenator. The syringe driver will be set to 0.6 mL/min (low dose propofol) or 1.2 mL/min (high dose propofol) resulting in a 6 mcg/ml or 12 mcg/ml supplementation of the blood cardioplegia (Figure 2). For the placebo supplementation (0.9% saline) the syringe driver will be set to 1.2 mL/min (same as high dose propofol).

Figure 2 demonstrates the method for administering the cardioplegia supplementation.

#### Anaesthetic regimen

To maintain consistent plasma concentrations of systemic propofol, anaesthetic management will adhere to a specified protocol (see supplementary material for details). The regimen will take into account the pharmacokinetics of propofol use for the induction and maintenance of anaesthesia and will be within usual practice. Data on anaesthetic regimen, including breaches, will be recorded.

#### Pre and post-operative management

The participant’s pre- and post-operative management will be in accordance with existing clinical protocols.

### Randomisation

The sequence of random allocations will be prepared in advance by an unblinded statistician using block randomisation, with varying block sizes and stratified by centre. To ensure allocation concealment; eligibility will be confirmed before randomisation can be carried out via a secure internet-based system. Participants will be randomly allocated in a 1:1:1 ratio to either low dose propofol, high dose propofol or placebo. Randomisation will be performed as close to surgery as possible by an unblinded member of the research team not involved in data collection. If a participant’s operation is unexpectedly cancelled, the allocation will be retained.

### Blinding

Study participants, the research and clinical care team (except the perfusionist), will be blinded to the treatment allocation. Blinding in the operating theatre was successfully achieved in the PROMPT study^10,11^ and similar methods will be used in the ProMPT2 study. The allocation details and materials required for the intervention (e.g. bag of saline or vial of propofol) will be handed to the perfusionist in a sealed opaque envelope. The infusion pump and line will be masked by a screen to avoid unblinding. The saline or propofol will be heavily diluted in the blood cardioplegia. Therefore, theatre staff should not be inadvertently unblinded.

Unblinding is only permitted if knowing the allocation will impact the treatment plan. If a request for unblinding occurs during surgery this will be facilitated by the perfusionist. After surgery, this will be managed by the unblinded member of the research team via the CRF or study databases. Any unblinding requests will be fully documented with a reason and will be monitored throughout the trial.

### Primary and secondary outcomes

The primary outcome will be myocardial injury, assessed by serial measurements of cTnT in serum from blood samples collected pre-operatively and during the first 48-hours after chest closure (see Table 1 for sampling schedule).

Secondary outcomes include;

(1) Systemic metabolic stress as measured by blood lactate;
(2) Renal function, as measured by creatinine in serum;
(3) Blood pH;
(4) Length of intensive care unit (ICU) stay;
(5) Length of postoperative hospital stay;
(6) Clinical outcomes and serious adverse events (SAEs), i.e. serious post-operative complications (e.g. myocardial infarction, permanent stroke, acute kidney injury) and death from any cause;
(7) Quality of life (QoL) measured using the Coronary Revascularisation Outcome Questionnaire (CROQ) and the EQ-5D-5L questionnaire.

The following secondary outcomes will be investigated in the Bristol cohort only;

(8) Markers of inflammation and oxidative stress as measured by tumour necrosis factor (TNF)-alpha, interleukin (IL)-10, IL-8, IL-6 and myeloperoxidase (MPO) in serum;
(9) The association between cTnT and circulating level of cardiac -released microRNA-1 and exosomal microRNA-1 content;
(10) The association between cTnT and microRNA and exosomal microRNA-1 content differs between groups (i.e. differs with the propofol supplementation received).

### Follow-Up

Participants will be followed up to hospital discharge and then at 3 and 12 months after randomisation.

### Data collection

A record of patient eligibility and approach will be kept. Data will be collected on paper case report forms (CRFs), entered onto a bespoke database and stored on a secure server. Access to the database will be via a secure password-protected web-interface. Data for the primary outcome and most of the secondary outcomes will be collected during the hospital stay.

QoL data will be collected pre-operatively (before randomisation) and at 3 and 12 months post-operatively. Participants will be able to complete follow-up questionnaires by post, online or by telephone. SAEs will be collected from start of surgery until hospital discharge.

The timing of blood sample collection for bio-marker outcomes is shown in Table 1. Where possible, cTnT will be measured by the local site hospital laboratory. If cTnT is not available at the site, cardiac troponin I (cTnI) will be measured for the monitoring of safety/toxicity. Serum will be stored locally for shipment to Bristol for cTnT measurement of the primary outcome.

### Monitoring safety and toxicity outcomes

Data on adverse events will be collected from the start of surgery throughout the participant’s post-operative hospital stay. This data will be monitored closely and serious adverse events will be reported on to the sponsor, REC and DMSC as required.

The ProMPT2 study will also assess safety/toxicity in the first 24 hours after surgery. Two safety/toxicity events are considered, one focusing on the heart and the other on the kidneys. The criteria used are;

(a) cTnT or cTnI concentration exceeding a threshold approximately 70 times the upper limit of normal (ULN) in serum from blood samples at 6 or 24 hours post chest closure (e.g. ULN for cTnT (Roche) is 14 ng/L and the threshold will be greater than 1000 ng/L).
(b) Creatinine concentration greater than or equal to 2 × baseline (pre-operative) in serum from blood sample at 24 hours post chest closure.

The criteria for reporting an “event” were based on past data^10,16^ and in discussion with clinicians on the study team. In the first PROMPT trial, 4/61 participants undergoing CABG surgery had a raised cTnT concentration >1000 ng/L and 1/61 had a raised creatinine ≥2 × baseline. The participant with a raised creatinine also had a raised cTnT. This gives an estimated incidence of 6.6% (and 1.6% for raised creatinine alone). The Titre-2 trial,^16^ which compared two thresholds for red cell transfusion after surgery, included 816 participants who underwent CABG surgery. Serial cTnT was not measured in this trial but creatinine levels were closely monitored. Overall, 13/807 participants had a raised creatinine ≥2 × baseline on day 1 after surgery, giving an incidence of 1.6%, which is consistent with the incidence in PROMPT.

A cumulative sum (CUSUM) chart is used to monitor early toxicity.^17,18^ The chart is updated as each participant is randomised. For safety purposes, participants are grouped according to the treatment received, rather than by the treatment intended. Beginning at zero, if the participant does not breach the criteria for a safety event, the cumulative sum remains unchanged. If an event occurs, one is added to the cumulative sum. The CUSUM chart includes thresholds, which correspond to an alert (lower threshold, lower dotted line) and an alarm (higher threshold, upper dotted line). The recommended chart parameters (i.e. the thresholds to signal alert and alarm) were determined by simulation, and agreed by the independent Data Monitoring and Safety Committee (DMSC) before adoption. It is crucial to select the optimal choice of threshold to minimise the number of events occurring before a true safety concern is detected while at the same time minimising false positive signals. The chart parameters agreed for the study will be 3 events (alert) and 5 events (alarm) per group. The DMSC will be notified if the CUSUM chart signals an alert or alarm. The identity of each group will only be revealed at the request of the DMSC.

**Figure 3:**
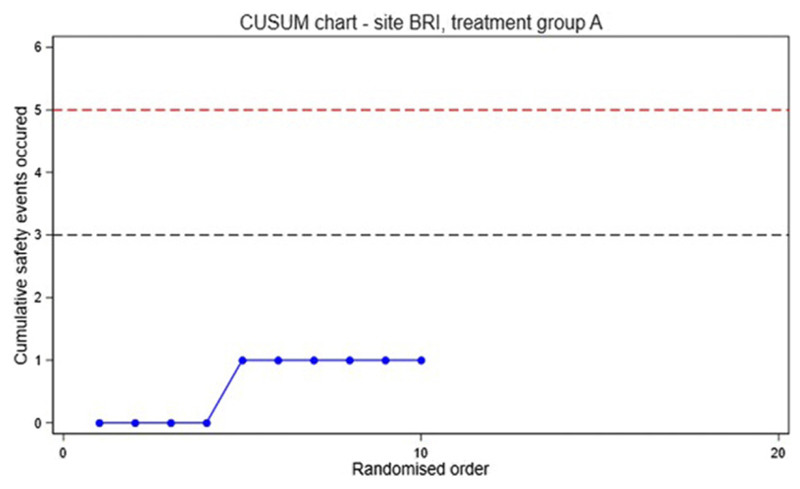
Example CUSUM chart. The first four participants randomised did not experience a safety event, the fifth participant did experience a safety event, so the cumulative sum increased to 1, the following 5 participants randomised did not experience a safety event. The lower dotted line represents an alert and the upper an alarm.

An example CUSUM chart is shown in Figure 3.

### Sample size calculation

The sample size assumptions underpinning the power calculation are based on cTnT levels which were measured in the first PROMPT RCT.^10^ Here we have assumed correlations between the pre and post intervention cTnT measures of 0.2 and correlations between successive post intervention measures of 0.7. Given these correlations and 5 repeated post-operative measures: a sample size of 240 participants (80 per group) will provide 90% power at a 5% significance level (2-sided) to detect a difference of 0.25 standard deviations (SD) in cTnT between adjacent groups (i.e. a difference of 0.25 SD between placebo and low dose propofol and between low and high dose propofol respectively), when considering all groups together on one overall analysis. This allows for 7.5% dropout. Additionally, the study will have 90% power to detect a difference of 0.5 SD (the target difference in the PROMPT trial) between any two groups at the 1.67% significance level (Bonferroni adjustment for three comparisons).

### Statistical analyses

Analyses will be performed on an intention-to-treat basis. Any non-adherence to the allocated group will be documented. The primary outcome (cTnT levels over the first 48 post-operative hours) will be analysed using a longitudinal mixed regression model, which allows for unbalanced data. The primary analysis will take place when follow-up is complete for all recruited participants. The value of including an interim analysis (e.g. to examine the dose-response relationship part way through the trial) will be discussed with the DMSC.

Secondary outcomes will be compared using logistic (binary variables), Cox proportional hazards (time-to-event variables), or linear mixed models (continuous variables measured at multiple time points) regression, with placebo supplementation as the reference group. Clinical outcomes (secondary outcome number 6) will be described but not formally compared, as event frequencies are expected to be too low to allow for a meaningful statistical comparison. The analyses of the association between cTnT and cardiac-released microRNA-1 and exosomal microRNA-1 content will be exploratory; the correlations between biomarkers will be investigated.

Analyses will be adjusted for baseline values (where appropriate) and centre (stratification factor). For each outcome, a model with indicators for the two propofol groups (i.e. assuming no ordering to the groups) will be compared with a model which assumes an ordinal linear dose response relationship with increasing propofol supplementation. An overall assessment of the effect of treatment across the three groups will be reported and differences between pairs of treatments will be quantified. Findings will be reported as effect sizes with 95% confidence intervals.

### Risk of bias

The following key features will help to minimise bias in the study;

(1) Selection/allocation bias will be minimised by concealing the randomisation allocation until sufficient information to uniquely identify the participant and confirm eligibility has been entered into the study database.
(2) Performance bias will be minimised by blinding participants, the research and clinical care team to the participant’s allocation.
(3) Detection bias will be minimised by blinding all individuals assessing outcomes and by using outcome measures that are defined as far as possible on the basis of objective criteria. Bio-chemical markers, including the primary outcome, will be analysed using standardised laboratory protocols by personnel blinded to the allocation.
(4) Bias due to missing outcome data (attrition bias) will be minimised by using methods to maximise the quality and completeness of the data and minimise treatment cross-overs (e.g. regular monitoring of data, detailed querying of data inbuilt into the study database, offering alternative methods for participating in follow-up (e.g. postal, online or telephone)). Data will be analysed by intention-to-treat (i.e. according to the treatment allocation, irrespective of future management and events), and every effort will be made to include all randomised participants. The primary outcome is measured within 48 hours after the operation when participants are still inpatients so missing data for the primary outcome is not anticipated.
(5) Reporting bias will be minimised by pre-specifying study outcomes and following a detailed analysis plan which will be prepared in advance of any comparative analyses of the study data.

### Patient and Public Involvement (PPI)

The proposed research was discussed with cardiac surgery patients in the design and funding stage, who were supportive. We will include patient and public involvement representation on the Trial Steering Committee (TSC) and will actively involve the PPI group for the duration of the study, including the dissemination of the results to participants and the public.

### Study management and oversight

The study is managed by the Bristol Trials Centre and sponsored by University of Bristol. The TSC is made up of representatives from the ProMPT2 study team and independent members approved by the funder. The DMSC consists of an independent medical statistician and medical experts in the field (again, approved by the funder). The TSC and DMSC meet as required; usually at least once a year.

### Ethics and Dissemination

The study received research ethics approval from South Central – Berkshire B Research Ethics Committee and MHRA in September 2018 (REC ref: 18/SC/0472). Participants have the right to withdraw at any time, and if so, will be treated according to standard hospital procedures. Participants who choose to withdraw will be able to continue participating in the study follow-up if they are willing.

We will present the study findings at international meetings and in peer reviewed publications. Additionally, social networking media will be used to disseminate and publicise study results. The study results will be disseminated to patients through patient organisations and newsletters to study participants.

### Changes to protocol since first regulatory approvals

Five substantial amendments to the study protocol have been made since first regulatory approvals (correct on 18th February 2022). The current version of the protocol is version 6.0 (dated 6^th^ November 2020):

(1) Amendment 1: (i) updated the anaesthetic regimen to reflect current practice and; (ii) updated the list of anticipated events associated with surgery.
(2) Amendment 2: (i) updated the eligibility criteria (it was noted that the pre-operative estimated glomerular filtration rate threshold was more stringent than required); (ii) allowed cTnI measurement for the monitoring of safety/toxicity outcomes as cTnT is not available at all participating centres; (iii) updated the SAE reporting period from the start of surgery and for the post-operative hospital stay (removing SAE reporting for 12 month follow up).
(3) Amendment 3: increased the number of participating sites.
(4) Amendment 4: (i) updated exclusion criteria to include; “Patients taking immuno-suppressants (e.g. methotrexate or azathioprine)” and; “Patients known to have cTnT level >500 ng/L (or cTnI level >600 ng/L) in the last 4 days (prior to eligibility check)”; (ii) updated the anaesthetic induction regimen to 0–2 mg/kg propofol as it was decided 0–1 mg/kg was too stringent. This remains within the normal range of clinical practice; (iii) updated the list of anticipated events associated with surgery.
(5) Amendment 5: (i) updated the recruitment and consent process to allow patients to give written informed consent via video or telephone call; (ii) added PICs to identify potentially eligible patients; (iii) allowed appropriately trained and qualified non-medical clinicians to review and sign study eligibility; (iv) updated the anaesthetic regimen.

### Study progress

Recruitment started in January 2019 and 182 participants have been recruited so far (correct on 18^th^ February 2022).

## Supplementary Material

Supplementary material

Appendix

## Figures and Tables

**Table 1:** Schedule of collection - biomarker outcomes.

Biomarker	Pre-op	10 mins post cross clamp release	Post chest closure				
			1 hr	6 hr	12 hr	24 hr	48 hr
Troponin T	**✓**		**✓**	**✓**	**✓**	**✓**	**✓**
Troponin I^a^	**✓**			**✓**		**✓**	
Lactate	**✓**	**✓**	**✓**	**✓**	**✓**	**✓**	**✓**
Creatinine	**✓**		**✓**	**✓**	**✓**	**✓**	**✓**
PH	**✓**		**✓**	**✓**	**✓**	**✓**	**✓**
Cardiovascular biomarkers (TNF-alpha, IL-6, IL-8, IL-10, MPO)^b^	**✓**		**✓**	**✓**	**✓**	**✓**	**✓**
microRNA-1 and exosomal microRNA-1^b^	**✓**		**✓**	**✓**	**✓**	**✓**	**✓**

a only at sites where cTnT measurement is not available.

b Bristol cohort only.

TNF = Tumour necrosis factor, IL = Interleukin, MPO = Myeloperoxidase.

## Data Availability

Data Storage. Study documentation will be retained in a secure location during the conduct of the study and 5 years after the end of the study, when all patient identifiable paper records will be destroyed by confidential means. To comply with the MRC Policy on Data Sharing, relevant “meta-data” about the study and the full dataset (without direct participant identifiers) will be held in-definitely on a secure server. A second file with a unique participant identifier, and key personal identifiers would also be retained indefinitely, but in a separate file and location, on a NHS hospital server. This will be retained for record linkage or similar for secondary research, subject to UK REC or other approved ethics review board review. Data Sharing. After trial completion and publication, the data generated from the trial will be available from the corresponding author on request. The request must include a pre-specified protocol describing the purpose, methods and analysis for the secondary research. The data may not be released unless all Bristol Trials Centre and Sponsor requirements are fulfilled (e.g REC approval).

## Summary

*Strengths and limitations of this study*

- First randomised controlled trial (RCT) to compare the safety and efficacy of different concentrations of propofol for myocardial protection during cardiopulmonary bypass.
- Novel use of cumulative sum (CUSUM) control chart to assess safety and toxicity.
- Blinding of all participants, the research and clinical care team to participant allocation (except perfusion staff for intervention delivery) will minimise performance and detection bias.
- The primary outcome (and majority of secondary outcomes) is objective and will be analysed using standardised laboratory protocols by personnel blinded to the allocation.
- The study only includes patients undergoing first-time, non-emergency, isolated coronary artery bypass surgery and uses a specific cardioplegia composition and anaesthetic regimen; which reduces its generalisability to the wider cardiac surgical population.
