# Supplementary material for: Efficacy of propofol-supplemented cardioplegia on biomarkers of organ injury in patients having cardiac surgery using cardiopulmonary bypass: A protocol for a randomised controlled study (ProMPT2)
Source: Perfusion. Author manuscript; Available in PMC 2025 Jan 7. (PMC7617284; doi:10.1177/02676591231157269)
Supplement: Supplementary material [file EMS200067-supplement-Supplementary_material.pdf]

SUPPLEMENTARY MATERIAL

1. Pharmacokinetics of propofol given in cardioplegia

During surgery, around 300-400ml of the blood/cardioplegia mixture will return to the circulation every 15 to 25 minutes. Assuming the patient has a circulating volume of 6l, this volume of cardioplegia solution will be diluted approximately 15 to 20-fold in the systemic circulation. The resultant rise in the circulating propofol concentration after each dose of cardioplegia will be very small. This equates to a rise in systemic propofol concentration of:

Low dose: 1800mcg total (6mcg/ml x 300ml) diluted in 6000ml circulation = 0.3mcg/ml

High-dose: 3600mcg total (12mcg/ml x 300ml) diluted in 6000ml circulation = 0.6mcg/ml

The half-life for the first dose of cardioplegia with propofol supplementation is short (1.6-4.0 minutes [42]). Approximately 4 to 6 half-lives (15 – 25 minutes) will elapse between each dose of cardioplegia. Therefore, the elevated propofol concentration in the systemic circulation will be close to zero before the next dose of cardioplegia is delivered.Thus, the systemic accumulation of propofol due to the cardioplegia supplementation will be negligible and the intended concentration in the cardioplegia will be the true concentration in the cardioplegia. From a safety perspective; this also means that the systemic concentrations of propofol will not rise above concentrations routinely attained during induction and maintenance of anaesthesia in surgery.

2. Standard cardioplegia composition and delivery

For all groups, blood cardioplegia with intermittent antegrade delivery will be used. This comprises of Potassium Chloride (KCl) (15%) 2mmol K+/ml and Magnesium Sulphate (MgSO4) (50%) 2mmol Mg2+/ml, mixed in a K+:Mg2+ 4:1 ratio. A 60ml syringe will be prepared with 20ml KCl and 5ml MgSO4 and will be loaded into a syringe driver. A roller pump will be used to draw oxygenated blood from the oxygenator and the K+/Mg2+ mixture will be added by a syringe pump downstream of the blood oxygenator.

3. Anaesthetic regimen

a) Induction: (i) 0 - 2.0mg/kg propofol, (ii) fentanyl OR alfentanil and (iii) a muscle relaxant;
b) Maintenance: (i) Isoflurane or sevoflurane in air/O2, (ii) plus additional opiate as required and (iii) Intravenous propofol infusion from the time of full heparinisation to maintain anaesthesia on bypass: 5 - 8mg/kg/hr
c) Post-operatively: (i) Propofol 2-7mg/kg/hr until extubation
