# Supplementary material for: Efficacy of propofol-supplemented cardioplegia on biomarkers of organ injury in patients having cardiac surgery using cardiopulmonary bypass: A protocol for a randomised controlled study (ProMPT2)
Source: Perfusion. Author manuscript; Available in PMC 2025 Jan 7. (PMC7617284; doi:10.1177/02676591231157269)
Supplement: Appendix [file EMS200067-supplement-Appendix.pdf]

Supplemental Material

Supplemental material for this article is available online.

References

1. Chambers DJ, Fallouh HB. Cardioplegia and cardiac surgery: pharmacological arrest and cardioprotection during global ischemia and reperfusion. *Pharmacol Ther* 2010; 127: 41–5220100414. DOI: 10.1016/j.pharmthera.2010.04.001
2. Vinten-Johansen J, Thourani VH. Myocardial protection: an overview. *J Extra Corpor Technol* 2000; 32: 38–48.
3. Halestrap AP, Pasdois P. The role of the mitochondrial permeability transition pore in heart disease. *Biochim Biophys Acta* 2009; 1787: 14021402–14021415. DOI: 10.1016/j.bbabio.2008.12.017
4. Chen Q, Camara AKS, Stowe DF, et al. Modulation of electron transport protects cardiac mitochondria and decreases myocardial injury during ischemia and reperfusion. *Am J Physiol Cell Physiol* 2007; 292: C137–C147. DOI: 10.1152/ajpcell.00270.2006
5. Halestrap AP. A pore way to die: the role of mitochondria in reperfusion injury and cardioprotection. *Biochem Soc Trans* 2010; 38: 841–860. DOI: 10.1042/BST0380841
6. Bovill JG. Intravenous anesthesia for the patient with left ventricular dysfunction. *Semin Cardiothorac Vasc Anesth* 2006; 10: 43–48. DOI: 10.1177/108925320601000108
7. Kamada N, Kanaya N, Hirata N, et al. Cardioprotective effects of propofol in isolated ischemia-reperfused guinea pig hearts: role of KATP channels and GSK-3beta. *Can J Anaesth* 2008; 55: 595–605. DOI: 10.1007/BF03021433
8. Xia Z, Godin DV, Chang TKH, et al. Dose-dependent protection of cardiac function by propofol during ischemia and early reperfusion in rats: effects on 15-F2t-isoprostane formation. *Can J Physiol Pharmacol* 2003; 81: 14–21. DOI: 10.1139/y02-170.
9. Lim KHH, Halestrap AP, Angelini GD, et al. Propofol is cardioprotective in a clinically relevant model of normothermic blood cardioplegic arrest and cardiopulmonary bypass. *Exp Biol Med (Maywood)* 2005; 230: 413–420. DOI: 10.1177/15353702-0323006-09
10. Rogers CA, Bryan AJ, Nash R, et al. Propofol cardioplegia: a single-center, placebo-controlled, randomized controlled trial. *J Thorac Cardiovasc Surg* 2015; 150: 1610–1619. DOI: 10.1016/j.jtcvs.2015.06.044
11. Plummer ZE, Baos S, Rogers CA, et al. The effects of propofol cardioplegia on blood and myocardial biomarkers of stress and injury in patients with isolated coronary artery bypass grafting or aortic valve replacement using cardiopulmonary bypass: protocol for a single-center randomized controlled trial. *JMIR Res Protoc* 2014; 3: e35. DOI: 10.2196/resprot.3353
12. Li J, Iorga A, Sharma S, et al. Intralipid, a clinically safe compound, protects the heart against ischemia-reperfusion injury more efficiently than cyclosporine-A. *Anesthesiology* 2012; 117: 836–846. DOI: 10.1097/ALN.0b013e3182655e73
13. Lou PH, Lucchinetti E, Zhang L, et al. The mechanism of Intralipid®-mediated cardioprotection complex IV inhibition by the active metabolite, palmitoylcarnitine, generates reactive oxygen species and activates reperfusion injury salvage kinases. *PLoS One* 2014; 9: e8720520140130. DOI: 10.1371/journal.pone.0087205
14. Huang H, Zhou C, Liu J, et al. Adding emulsified isoflurane to cardioplegia solution produces cardiac protection in a dog cardiopulmonary bypass model. *Sci Rep* 2016; 6: 23572–20160428. DOI: 10.1038/srep23572
15. Calafiore AM, Teodori G, Mezzetti A, et al. Intermittent antegrade warm blood cardioplegia. *Ann Thorac Surg* 1995; 59: 398–402. DOI: 10.1016/0003-4975(94)00843-v
16. Reeves BC, Pike K, Rogers CA, et al. A multicentre randomised controlled trial of transfusion indication threshold reduction on transfusion rates, morbidity and health-care resource use following cardiac surgery (TITRe2). *Health Technol Assess* 2016; 20: 1–260. DOI: 10.3310/hta20600
17. Steiner SH, Cook RJ, Farewell VT. Risk-adjusted monitoring of binary surgical outcomes. *Med Decis Making* 2001; 21: 163–169. DOI: 10.1177/0272989X0102100301
18. Rogers CA, Reeves BC, Caputo M, et al. Control chart methods for monitoring cardiac surgical performance and their interpretation. *J Thorac Cardiovasc Surg* 2004; 128: 811–819. DOI: 10.1016/j.jtcvs.2004.03.011

Appendix

Abbreviations

AVR	Aortic valve surgery
BTC	Bristol Trials Centre
CABG	Coronary artery bypass grafting
CPB	Cardiopulmonary bypass
CRF	Case report form
CROQ	Coronary revascularisation outcome questionnaire
cTnI	Cardiac troponin I
cTnT	Cardiac troponin T
DMSC	Data monitoring and safety committee
EME	Efficacy and Mechanism Evaluation
EQ-5D-5L	EQ-5D-5L quality of life questionnaire
GCP	Good Clinical Practice
ICU	Intensive care unit
IL	Interleukin
LV	Left ventricular
MHRA	Medicines and healthcare products regulatory agency
MPO	Myeloperoxidase
NaCl	Sodium chloride
NIHR	National Institute for Health Research
PPI	Patient and Public Involvement
PICs	Participating Identification Centres
PIL	Patient information leaflet
QoL	Quality of Life

RCT	Randomised controlled trial
ROS	Reactive oxygen species
REC	Research ethics committee
RPI	Reperfusion injury
SAE	Serious adverse event
SAR	Serious adverse reaction
SD	Standard deviations
SCTS	Society of the Cardiothoracic Surgeons of Great Britain and Ireland
SPCS	Society of Clinical Perfusion Scientists of Great Britain and Ireland
SIRS	Systemic inflammatory response syndrome
SmPC	Summary of product characteristics
SOP	Standard operating procedure
TNF	Tumour necrosis factor-alpha
TSC	Trial steering committee
ULN	Upper limit of normal.
